# The Cardiac Electrophysiology Web Lab

**DOI:** 10.1016/j.bpj.2015.12.012

**Published:** 2016-01-19

**Authors:** Jonathan Cooper, Martin Scharm, Gary R. Mirams

**Affiliations:** 1 Department of Computer Science, University of Oxford, Oxford, United Kingdom; 2 Department of Systems Biology and Bioinformatics, University of Rostock, Rostock, Germany

## Abstract

Computational modeling of cardiac cellular electrophysiology has a long history, and many models are now available for different species, cell types, and experimental preparations. This success brings with it a challenge: how do we assess and compare the underlying hypotheses and emergent behaviors so that we can choose a model as a suitable basis for a new study or to characterize how a particular model behaves in different scenarios? We have created an online resource for the characterization and comparison of electrophysiological cell models in a wide range of experimental scenarios. The details of the mathematical model (quantitative assumptions and hypotheses formulated as ordinary differential equations) are separated from the experimental protocol being simulated. Each model and protocol is then encoded in computer-readable formats. A simulation tool runs virtual experiments on models encoded in CellML, and a website (https://chaste.cs.ox.ac.uk/WebLab) provides a friendly interface, allowing users to store and compare results. The system currently contains a sample of 36 models and 23 protocols, including current-voltage curve generation, action potential properties under steady pacing at different rates, restitution properties, block of particular channels, and hypo-/hyperkalemia. This resource is publicly available, open source, and free, and we invite the community to use it and become involved in future developments. Investigators interested in comparing competing hypotheses using models can make a more informed decision, and those developing new models can upload them for easy evaluation under the existing protocols, and even add their own protocols.

## Introduction

Mathematical and computational modeling of cardiac electrophysiology has a long history (1, 2). Encoding hypotheses about how systems work in a quantitative form has yielded valuable insights into cellular behavior and the roles of different ionic currents (3), the mechanisms behind arrhythmias (4, 5), and treatments such as defibrillation (6). As is the case with mathematical modeling in general, models are developed to represent specific quantitative hypotheses and to answer specific scientific questions. Therefore, studies published about new models display behavior under particular experimental conditions and draw inferences from that behavior. This is, of course, appropriate and useful.

However, this approach can also be limiting. If we consider that a mathematical model is a quantitatively encoded hypothesis (or set of hypotheses), how can we see which hypothesis is best supported by new data? One group of researchers may have the ability to compare how their own models behave in a wide range of different situations (7), or easily vary their simulations to represent the different experimental scenarios. Nevertheless, there is no automated solution for examining how a particular model behaves under a range of experimental conditions, let alone for comparing the behaviors of any of the published models. As a result of this technical barrier, only a very few published studies have compared models/hypotheses (with rare exceptions (e.g., 8, 9, 10, 11, 12)). The need will become particularly acute as models begin to be used in simulation studies for applications such as drug safety testing (7, 13, 14, 15), where we rely on behavioral predictions beyond the normal regime in which many models were originally developed and tested.

The challenge has its roots in the publication medium. Traditionally, publishing a model involves displaying the model equations, originally implemented within some computational software, in print form. This makes them difficult to reproduce or extend, and indeed this is often impossible (e.g., due to missing information or typographical errors) without assistance from the original author(s) (16, 17). Releasing the source code for the original model implementation helps, and has been done by many groups. However, comparing models available in this form is still extremely challenging, as they have been written in different programming languages, for different computational platforms, and in different formats, and are not readily interoperable.

An excellent effort has been made to encode many of the action potential (AP) models in the CellML format (18, 19, 20), a symbolic definition of the mathematical model equations that is independent of any particular numerical method or simulator software, and is designed to enable easy manipulation of the model’s mathematics. This enables the model equations (i.e., systems of ordinary differential equations) to be shared unambiguously, and code for particular programming languages can be autogenerated from the CellML format.

However, despite more than 50 years of cardiac modeling, and now the availability of hundreds of models and variants for cardiac electrophysiology, investigators have had nowhere to look up simple model characteristics such as the AP waveform at a given pacing rate. There has been no automatic mechanism for checking even the published behaviors ascribed to a model, let alone other potential or expected capabilities. Given this, it is unsurprising that occasionally the curated model descriptions do not match the original implementations, and we give one example of this further below.

We believe a better way forward is provided by the concept of virtual experiments (21), the in silico analogs of wet lab experiments, defined by protocols that crucially can be encoded in a form amenable to processing by a computer program, and applied to different models of a system. This could be seen as an analog of an experimental protocol that different labs could follow to reproduce research findings, which is increasingly being recognized as essential for experimental research (22). In earlier work (23), we described how implementing this concept in tools for the functional curation of models could address some of the challenges described above. In particular, we claimed that being able to examine how models behave in different experimental scenarios would help to guard against the potential misuse of models, which could otherwise be encouraged by their already-easy availability.

Here, we present the Cardiac Electrophysiology Web Lab, a user-friendly web interface that allows modelers to characterize their (and others’) cardiac electrophysiology models, provided they are encoded using CellML, and to compare a model’s behavior against that of any other model under a wide range of simulated experimental conditions. It must be emphasized that the Web Lab *does not* make any judgments as to whether the models behave appropriately in a given experiment, or which is best. Instead, it provides a system to enable careful comparison and analysis of the behavior of models in multiple virtual experiments.

## Materials and Methods

**Figure 1:**
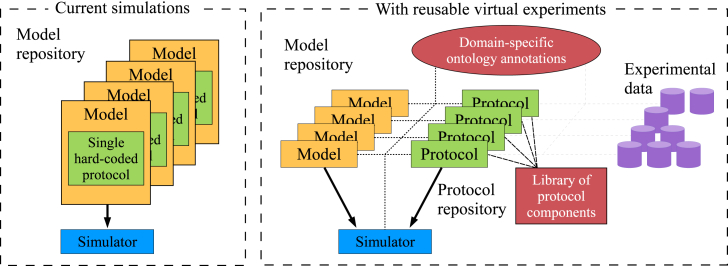
Schematic of the technical infrastructure underlying our website. In the state-of-the-art model repositories, each available model description is actually a model of a particular experimental setup (generally 1 Hz pacing in cardiac AP models). In our database, models represent a biological system, and experimental protocols are described separately and may be applied to any model. Experimental data will be directly comparable with the results of certain protocols. To see this figure in color, go online.

To automatically characterize and compare the behavior of models in different experimental scenarios, both the models and the protocols must be described in formats that can be understood by our software, rather than as black-box programs in their own right. In addition, the details of the protocol need to be separated from the model equations, thus moving us from models of a particular experiment to models of a biological system. Different protocols may then be applied to each model of the system, exercising them in different ways. This approach is shown schematically in Fig. 1.

We utilize the existing CellML format (18) to encode the model descriptions, and our tool can simulate any model that mathematically is a system of ordinary differential equations (including the trivial case of a purely algebraic model). The model and protocol descriptions, as well as the results of virtual experiments, are packaged into single files using the COMBINE Archive format (24). Although we are contributing to the development of a community standard format for protocol descriptions (i.e., the Simulation Experiment Description Markup Language (SED-ML) (25)), it does not yet meet all our requirements. In the interim, we have developed our own extensions to this language (23), with a text syntax that facilitates understanding and editing of the protocols (26). The techniques required for running experiments using any protocol on any model are detailed in our earlier publications (23, 27). The main features are the use of annotations indicating the physiological meaning of model variables, to avoid confusion over naming (these can be added to the CellML files within minutes in the Web Lab), and automated unit conversions to ensure mathematically consistent simulations. These simulation tools are built on top of the Chaste libraries for computational biology (28, 29, 30).

The tools are made available to the user via a web interface to provide an installation-free, interactive experience. Behind the scenes, a database stores model and protocol descriptions along with the cached results of the corresponding experiments (every protocol has been run on every compatible model, i.e., every model that contains the biological quantities being probed by the protocol). These descriptions and results may be viewed by anyone, with plots of the results rendered in the web browser. In addition, any experiments may be compared, combining their results in a single graphic.

Note that these virtual experiments are not performed on the fly when results are viewed. Some protocols require extensive simulation and postprocessing, and thus take a nontrivial length of time to run. Instead, experiments are run by the Web Lab when new (or updated) models or protocols are added to the system, and the results are cached.

Although anonymous users may browse and compare these stored results, it is also possible to sign up for an account and receive permission to add your own models and protocol descriptions; these may be kept private or published for all to see. Registered users may therefore run new experiments on our servers, and if the corresponding model and protocol have been made public, the results will also be visible to all. The underlying simulation environment and the Web Lab portal code have been released as open source and can be accessed via the web portal, as can the documentation on using the system, uploading your own models, and writing your own protocols.

## Results

**Figure 2:**
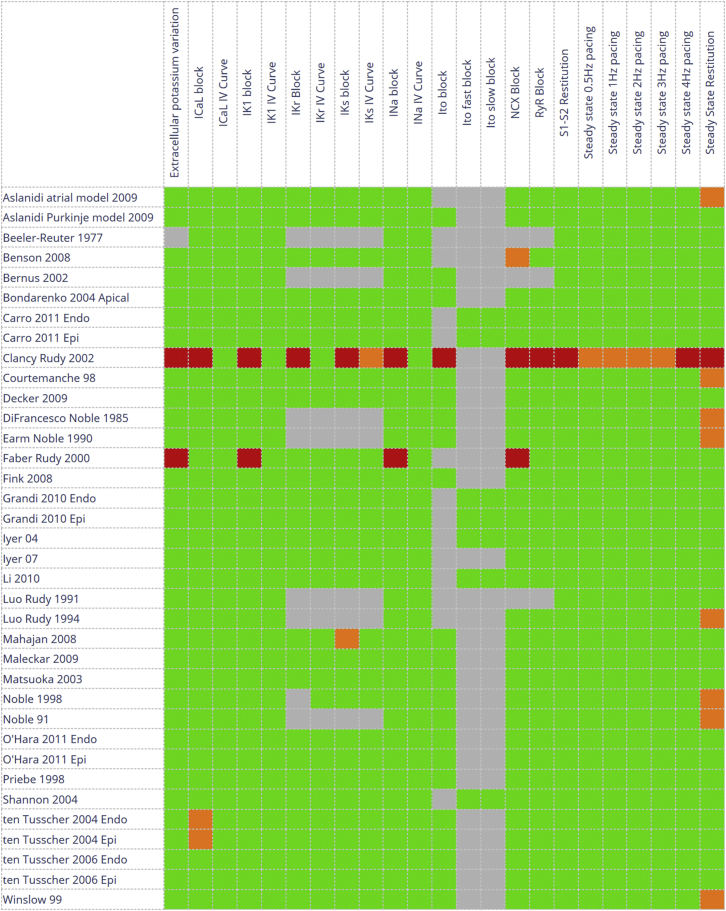
Overview of the virtual experiments available in our system at the time of this writing (see https://chaste.cs.ox.ac.uk/FunctionalCuration/db.html for the current status). Each square represents the stored results of a single virtual experiment, color coded according to status. Green indicates that the protocol ran to completion, orange that it did not complete but some of the expected graphs are nevertheless available (so only a subset of the simulations and/or postprocessing failed), red that no graphs are available, and gray that the model and protocol are incompatible (i.e., the model does not contain some biological feature probed by the protocol). Shades of blue indicate a queued or running experiment (no examples shown). Note, therefore, that the colors do not indicate model correctness in any sense. To see this figure in color, go online.

In this section, we showcase some of the results that are already available online to provide an impression of the potential uses, capability, and flexibility of the Web Lab. Fig. 2 displays the experiment overview table. Results are color coded according to the experiment’s state, i.e., queued, running, inapplicable (the protocol’s required quantities are not present, or not labeled, in the model), failed to run (usually due to numerical instabilities; see below), partially finished (some postprocessing was not possible), or successfully finished. Note that we do not compare simulated results against experimental data, and hence the color coding *does not* represent model correctness or agreement with experimental data in any sense; it simply indicates the degree to which the simulation experiment was able to be run. Accordingly, a model displaying all green results should not be considered as the best model.

Below, we show some results of individual virtual experiments, highlighting the ways in which different models (or different hypotheses) can make very different predictions. This illuminates certain areas that will require careful attention in cardiac electrophysiology modeling.

### Exploring model characteristics

**Figure 3:**
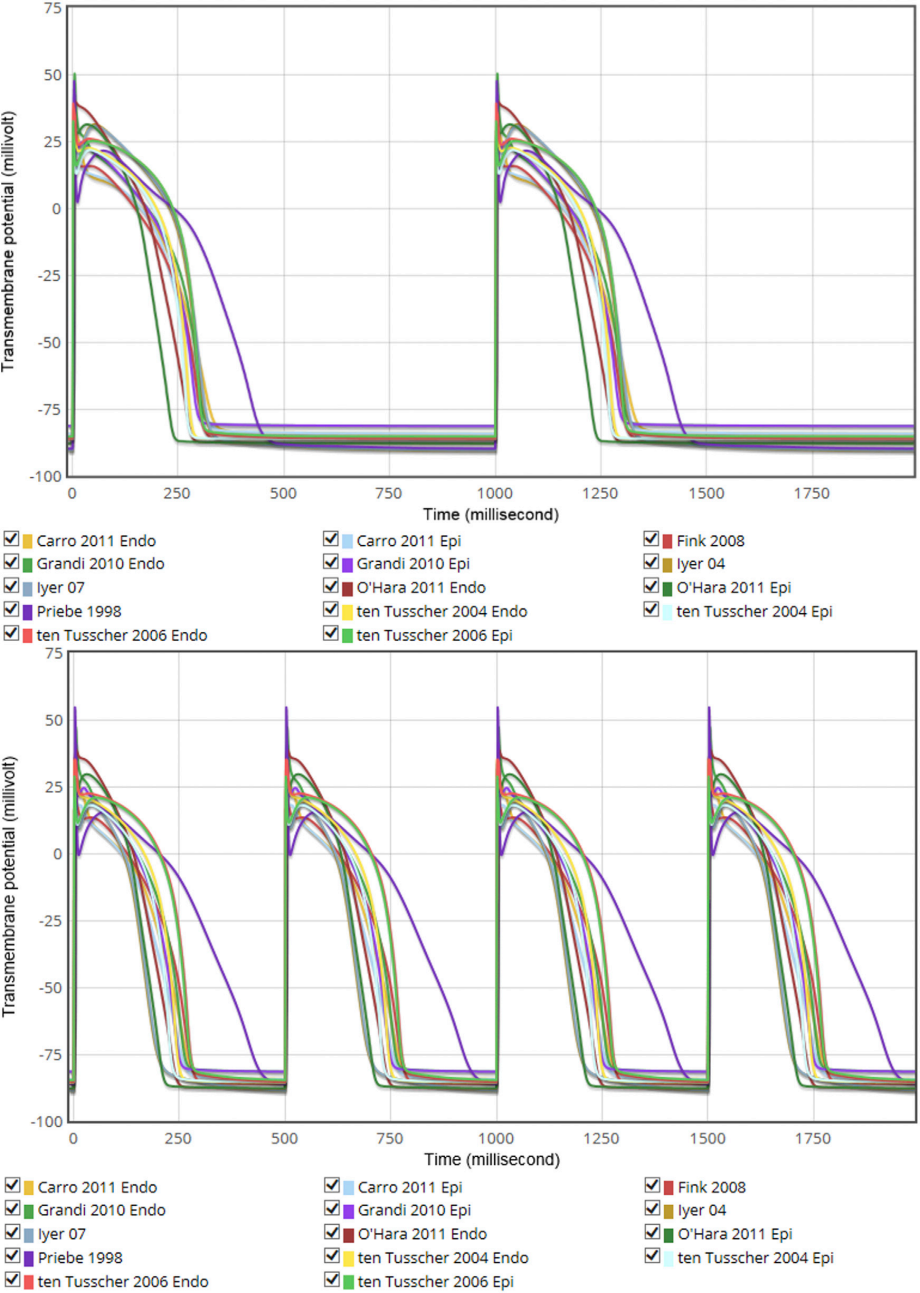
1 Hz (*top*) and 2 Hz (*bottom*) steady-pacing AP waveforms for a selection of human ventricular cell models. See https://chaste.cs.ox.ac.uk/q/2015/fc/fig3a and https://chaste.cs.ox.ac.uk/q/2015/fc/fig3b for the Web Lab originals. To see this figure in color, go online.

For the first time cardiac electrophysiology researchers can easily examine the AP waveforms produced by different models. In Fig. 3 we present a snapshot of APs for several human ventricular models at both 1 Hz and 2 Hz. Note that these voltage traces are not the only outputs produced by the corresponding protocol: one can view and compare other outputs on the live system by selecting the appropriate Action icons below the plots. For instance, the calcium transients corresponding to the APs in Fig. 3 can be compared at https://chaste.cs.ox.ac.uk/q/2015/fc/Ca1Hz and https://chaste.cs.ox.ac.uk/q/2015/fc/Ca2Hz.

**Figure 4:**
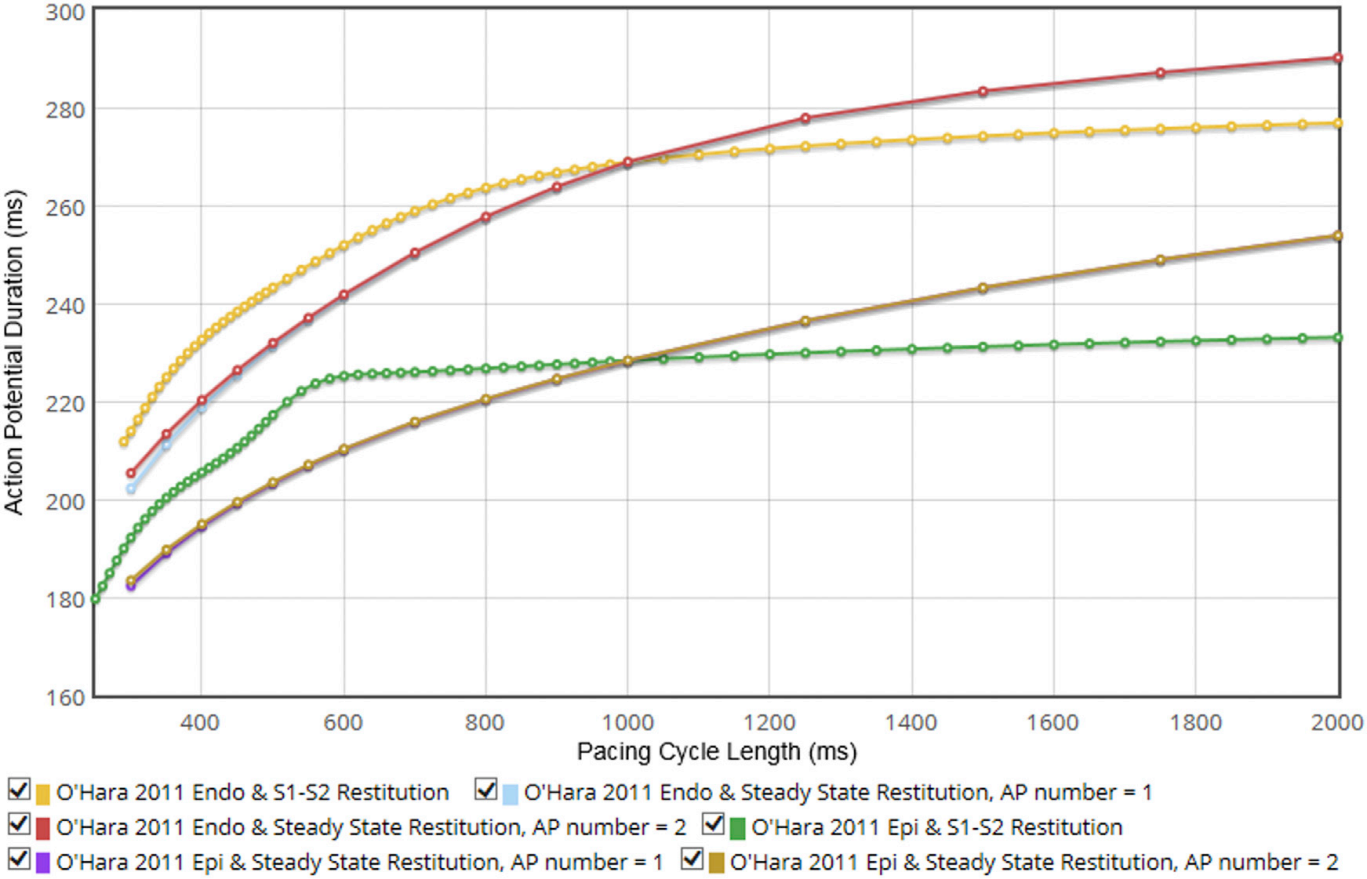
Restitution curves for the O’Hara 2011 model epi- and endocardial variants. Variation in APD at 90% repolarization is shown for the S1-S2 protocol with the initial stimulus interval S1 set to 1000 ms, and for steady-state restitution (in which two paces are analyzed and plotted as two lines, to show fork or alternans at short rates, visible in the endocardial variant). This demonstrates the Web Lab’s ability to run complex protocols with intricate postprocessing. See https://chaste.cs.ox.ac.uk/q/2015/fc/fig4 for the Web Lab original. To see this figure in color, go online.

One can easily encode more complex protocols (e.g., S1-S2 or steady-state restitution curves) and compare model behaviors under these protocols, as shown in Fig. 4 for the O’Hara 2011 model (31) epi- and endocardial variants.

**Figure 5:**
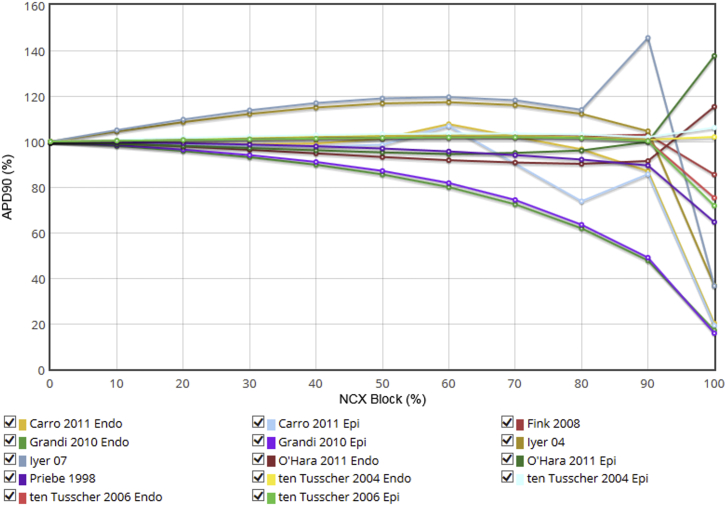
Effect of blockade of NCX on steady-state APD in some human ventricular cell models. Note that across 0–80% NCX block, some models predict little effect (<5% change), whereas others predict 20% prolongation and still others predict 20% shortening. At 80–100% block, the results vary dramatically, with models predicting effects ranging from 45% prolongation to 20% shortening compared with control. See https://chaste.cs.ox.ac.uk/q/2015/fc/fig5 for the Web Lab original. To see this figure in color, go online.

Although a large number of models include dynamic changes in ionic concentrations (first introduced by DiFrancesco and Noble (32) in 1985), ionic homeostasis would appear to be one of the more controversial areas, as evidenced by the wide variety of model responses (or hypothesis predictions) to alterations of this system. For example, in Fig. 5 we present the (steady-state) effect on the AP duration (APD) of progressive block of the sodium-calcium exchanger (NCX), implemented by scaling its maximum current density. The models make a wide range of predictions, reflecting the current limitations of our knowledge regarding intracellular sodium and calcium homeostasis (33), and therefore an appropriate model for any study involving changes to NCX conductance should be selected carefully. The Web Lab can assist in this by demonstrating how different models behave.

We have already used the Web Lab to examine recent human ventricular models under drug-induced blockade of certain ion channels, using a conductance-block model (34) rather than introducing models for the kinetics of particular drug-ion channel interactions. These data formed part of a recent study (35), making this part of the study quick to produce, immediately replicable, and trivial to extend should a novel model be produced (or an existing model be updated). Although the protocols currently on the site look at single-channel effects in isolation, the protocol language is rich enough to explore multichannel effects as well.

The model behaviors we have highlighted here are the tip of the iceberg, and are simply intended to give an impression of the power of the approaches that the Web Lab enables. Tutorial materials prepared for a recent workshop (see below) and our previous publications (23, 26) give more information about the possibilities for protocol descriptions.

### Correcting errors in model encodings

A discussion about the results of the Decker 2009 model S1-S2 restitution curve (as published in our pilot study (23)) with the senior author, Prof. Y. Rudy, led us to a careful comparison of our results with those in their original model publication (36). The differences uncovered an error in the CellML implementation of the Decker model, which had been available since March 2010 (for full details, see http://mirams.wordpress.com/2013/10/22/importance-of-curating-models/). The CellML file was corrected and is now providing an accurate representation of the model to the community (https://chaste.cs.ox.ac.uk/q/2015/fc/s1s2). Differences between the original and corrected model versions can be displayed in the Web Lab by using the model-comparison tool BiVeS (37, 38) (see https://chaste.cs.ox.ac.uk/q/2015/fc/diff for the results). These differences only become apparent when a model is tested in a range of situations, which the Web Lab enables.

### Steady states

**Figure 6:**
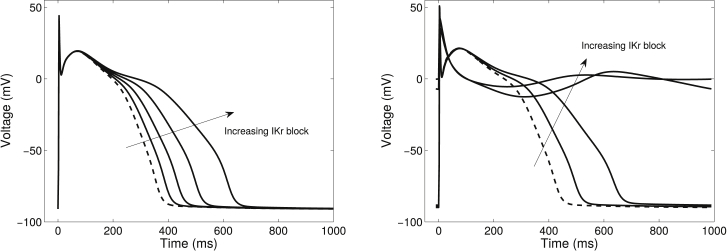
Effect of examining behavior before and after steady state is reached, for 0% (*dashed line*), 25%, 50%, 75%, and 100% block of the rapid delayed rectifier potassium current (IKr) in the Priebe 1998 model. Left: after just one pace at each degree of IKr block, the results are the same as those shown in Priebe and Beuckelmann (39). Right: the same model after 10,000 paces for each degree of IKr block as shown in the Web Lab. Note that even the control AP varies considerably, and is much longer in the steady-state case.

Deterministic electrophysiology models typically tend toward a limit cycle behavior at a given pacing rate. Many of our protocols examine interventions at this steady state rather than after a limited number of paces. In some models, we have observed behavior that is either not the same as that previously described (the Priebe 1998 model (39)) (Fig. 6) or seems nonphysiological (the Aslanidi 2009 atrial model (40)), suggesting that the model equations, or their initial conditions, may require alteration. Nonphysiological steady states can often be attributed to drift in ionic concentrations due to imbalance when not all currents are accounted for in concentration equations (41). It is useful to be able to distinguish those models that are reaching a limit from those that are continuing to drift, as the latter should not be used in simulations that examine steady-state behavior or run for a long time.

## Discussion

We have presented a new online resource for users and developers of mathematical models of cardiac electrophysiology. As shown in the previous section, it offers great flexibility for analyzing and comparing models under different experimental conditions. This will help model users to select suitable models for their simulation studies by ensuring that relevant basic behavior can be reproduced (for instance, that a model intended for use in simulating arrhythmia has suitable restitution properties). It can also highlight models whose implementations have problems with numerical stability, or those that drift to nonphysiological regimes.

Although we have highlighted some interesting results that arose from previous experiments, we deliberately did not try to extract all publishable comparisons from these data before making them available. Instead, we preferred to concentrate on producing a usable public resource for the benefit of the community. Therefore, we welcome and invite others to examine the results for themselves, add new models, write code for new protocols, and explore what they find.

Despite our efforts to produce reliable virtual experiments with this system, unexpected behavior may occur that is not necessarily a real consequence of the model. Mathematical singularities or other numerical simulation issues may cause the simulated experiment to fail, leading to many of the red boxes in Fig. 2. Sometimes the published representation of the model is in error, or its CellML encoding is (as was the case for the Decker 2009 model discussed above). On other occasions, the protocol, especially the postprocessing section, may need further refinement to account for raw simulation results that fall somewhat outside the expected regime—computing a robust APD that accounts for any shape of AP, particularly pathological cases, is surprisingly complex. We invite readers who find any examples of behavior that does not match other model implementations to contact us and we will attempt to determine the cause. This may, of course, result from a deficiency in our software, although we have an extensive bank of automated software tests to guard against this. In addition, since all the methods and software needed to reproduce results shown in the Web Lab are openly available, the community can examine how the results were produced in full detail.

Our online resource will also be of benefit to model developers. They may upload their in-development models to the system, keeping them private if needed, to evaluate their behavior against a much wider range of protocols than are typically considered during construction of a new model. If a particular experiment is not already available, the corresponding protocol may be submitted as well. New model versions may be uploaded until the desired set of behavioral characteristics is obtained, and the final model can be made public when it is published. The publication could even refer to the stored results as evidence that the model has been thoroughly tested.

Notwithstanding the considerable utility of the existing system, there are many aspects that will require further development as this becomes a hub for researchers working with cardiac electrophysiology models. We particularly encourage users to contribute new models and protocols, and we held an initial training workshop in September 2015 at which 25 researchers learned how to do this (and provided valuable feedback on the system). The materials developed for this workshop are now linked from the Web Lab. We aim to provide a protocol editor to ease this task for those without programming experience. In the meantime, users are welcome to suggest new protocols, and we will assist with encoding them.

As the Web Lab becomes a community resource, various challenges will emerge. Users will have questions about why particular behaviors arise. Incorrectly encoded or annotated models may be uploaded and produce results that do not reflect the published model, resulting in potential reputational damage. Managing queries and user support would require a significant time commitment if handled solely by the Web Lab developers. Instead, we envisage the user community becoming self-supporting. There is already a users’ mailing list, but one potential avenue is to integrate moderated discussion forums with the Web Lab. Threads could then be linked to individual models or results, and explanations or suggestions contributed. This would be particularly important for giving model authors a right of reply. A similar moderation approach could also be used before models or protocols are allowed to be made public. We will also, whenever possible, ensure that the corresponding author of a published model is emailed before a version of that model is made public on the Web Lab.

As noted above, we use annotations indicating the physiological meaning of model variables to form the interface between models and protocols, so that a single protocol can be applied to models that may use different names to represent the same concept. In our database, we thus include copies of models from the CellML repository (20) annotated with terms developed for this purpose. Ideally, these annotations would instead be stored along with the reference versions of the model in the CellML Physiome Model Repository itself, and community-accepted annotations would be used to promote wider interoperability. The direct use of curated models would also address the accountability challenge mentioned above. Related ongoing work is adding more structure to our annotations by defining relationships between terms. This structure could then be used by enhanced tools to provide even more sophisticated interfacing between models and protocols, for instance, by clamping all extracellular concentrations without having to specify which ions may be present in the model.

Other enhancements to the tools, and indeed the protocol language, may also be required as new ideas for protocols arise. We are looking at incorporating parameter-estimation techniques into this framework, and further automated checking of experiment results could also be investigated (42). It would also be desirable to use a community-accepted standard for protocols rather than our own representation, and therefore we are proposing features of our new language for incorporation into future versions of the SED-ML standard being developed by the systems biology community (25).

Finally, the most important ingredient that is missing in our current implementation is a direct link to experimental data. Since protocol descriptions should represent experiments that can be performed in a wet lab (43), it is natural to associate corresponding experimental data sets with each protocol. Simulated experimental results could then be compared automatically against these data sets, to reveal the extent to which different models match our current knowledge of the system. Although public data repositories for electrocardiogram data exist (e.g., PhysioNet, www.physionet.org (44)), there is a notable lack of open sources for cell-level electrophysiology data, which could become a serious impediment to progress. Eventually, we envisage that the model descriptions could be associated explicitly with all of the data that were originally used to parameterize them. As new data become available, all relevant models could be validated against them, and even reparameterized automatically to capture the latest experimental results within a quantitative model (21).

## Author Contributions

J.C. and G.R.M. devised the system. J.C. wrote the simulation software. M.S. and J.C. wrote the web interface. G.R.M. and J.C. analyzed the results. All authors contributed to writing the manuscript and approved the final version.
